# Geographical Accessibility to Glucose-6-Phosphate Dioxygenase Deficiency Point-of-Care Testing for Antenatal Care in Ghana

**DOI:** 10.3390/diagnostics10040229

**Published:** 2020-04-16

**Authors:** Desmond Kuupiel, Kwame M. Adu, Vitalis Bawontuo, Duncan A. Adogboba, Paul K. Drain, Mosa Moshabela, Tivani P. Mashamba-Thompson

**Affiliations:** 1 Department of Public Health Medicine, School of Nursing and Public Health, University of KwaZulu-Natal, Durban 4041, South Africa; Moshabela@ukzn.ac.za (M.M.); Mashamba-Thompson@ukzn.ac.za (T.P.M.-T.); 2 Research for Sustainable Development Consult, Sunyani, Ghana; bawontuovitalis@yahoo.com; 3 Department of Geography, University of Ghana, Legon, Ghana; meous007@gmail.com; 4 Faculty of Health and Allied Sciences, Catholic University College of Ghana, Fiapre, Sunyani, Ghana; 5 Regional Health Directorate, Ghana Health Service, Upper East Region, Bolgatanga, Ghana; alemna@gmail.com; 6 Department of Global Health, University of Washington, Seattle, WA 98195, USA; pkdrain@uw.edu; 7 Department of Public Health, Faculty of Health Sciences, University of Limpopo, Polokwane 0723, South Africa

**Keywords:** geographical access, glucose-6-phosphate dioxygenase deficiency, point-of-care testing, antenatal care, upper east region, Ghana

## Abstract

Background: Glucose-6-Phosphate Dehydrogenase (G6PD) deficiency screening test is essential for malaria treatment, control, and elimination programs. G6PD deficient individuals are at high risk of severe hemolysis when given anti-malarial drugs such as primaquine, quinine, other sulphonamide-containing medicines, and chloroquine, which has recently been shown to be potent for the treatment of coronavirus disease (COVID-19). We evaluated the geographical accessibility to POC testing for G6PD deficiency in Ghana, a malaria-endemic country. Methods: We obtained the geographic information of 100 randomly sampled clinics previously included in a cross-sectional survey. We also obtained the geolocated data of all public hospitals providing G6PD deficiency testing services in the region. Using ArcGIS 10.5, we quantified geographical access to G6PD deficiency screening test and identified clinics as well as visualize locations with poor access for targeted improvement. The travel time was estimated using an assumed speed of 20 km per hour. Findings: Of the 100 clinics, 58% were Community-based Health Planning and Services facilities, and 42% were sub-district health centers. The majority (92%) were Ghana Health Service facilities, and the remaining 8% were Christian Health Association of Ghana facilities. Access to G6PD deficiency screening test was varied across the districts, and G6PD deficiency screening test was available in all eight public hospitals. This implies that the health facility-to-population ratio for G6PD deficiency testing service was approximately 1:159,210 (8/1,273,677) population. The spatial analysis quantified the current mean distance to a G6PD deficiency testing service from all locations in the region to be 34 ± 14 km, and travel time (68 ± 27 min). The estimated mean distance from a clinic to a district hospital for G6PD deficiency testing services was 15 ± 11 km, and travel time (46 ± 33 min). Conclusion: Access to POC testing for G6PD deficiency in Ghana was poor. Given the challenges associated with G6PD deficiency, it would be essential to improve access to G6PD deficiency POC testing to facilitate administration of sulphadoxine-pyrimethamine to pregnant women, full implementation of the malaria control program in Ghana, and treatment of COVID-19 patients with chloroquine in malaria-endemic countries. To enable the World Health Organization include appropriate G6PD POC diagnostic tests in its list of essential in-vitro diagnostics for use in resource-limited settings, we recommend a wider evaluation of available POC diagnostic tests for G6PD deficiency, particularly in malaria-endemic countries.

## 1. Introduction

Glucose-6-phosphate dehydrogenase (G6PD) deficiency is a sex-link hereditary mutation on the X-chromosome [1,2,3]. G6PD is an enzyme that helps protect red blood cells from damage and premature destruction [2,3]. Although most G6PD deficiency patients do not show clinical signs and symptoms, it is commonly characterized by abnormally low levels of G6PD, and some variants could be fatal due to complete loss of enzyme activity [2,3]. Globally, G6PD deficiency affects approximately 400 million people and its prevalence is highest particularly, in malaria-endemic countries ranging from 5 to 24% [4,5]. A systematic review in 2009 showed that Sub-Saharan African (SSA) countries accounted for the highest G6PD prevalence before and after adjusting for the assessment method [4]. The meta-analysis also showed a high degree of geographical heterogeneity of G6PD prevalence estimates, which seemed to be due to differences in G6PD deficiency assessment and diagnostic procedures [4]. The magnitude and geographical (global, regional, and country-level) heterogeneity of G6PD deficiency prevalence rates have public health implications, especially in malaria control or elimination programs involving the administration of antimalarial medicines for most malaria-endemic countries such as Ghana. G6PD deficient individuals are at high risk of severe hemolysis when given anti-malarial drugs such as primaquine, quinine, other sulphonamide-containing medicines [6,7,8], and chloroquine which has been shown to be potent for the treatment of coronavirus disease (COVID-19) [6,9,10], These drugs may cause an irreversible oxidative activity of the body’s metabolites on erythrocytes [7,8].

Ghana is at risk of malaria throughout the year with over 21% of malaria parasitemia of which approximately 98% result from Plasmodium falciparum infection [11,12]. Ghana is ranked fourth alongside Burkina Faso, Uganda, and accounted for 4% of the world’s 219 million malaria cases in 2017 according to a World Health Organization (WHO) 2018 report [13,14]. Malaria infection during pregnancy has substantial risk for the mother, her fetus and the newborn such as placental malaria infection, low birth weight, and severe maternal anemia [15,16,17,18,19,20]. As part of the intermittent preventive treatment during pregnancy (IPTp) in Ghana, a single of sulphadoxine-pyrimethamine (SP) is administered to pregnant women at predefined intervals after quickening (16 gestational weeks) to reduce malaria parasitemia and poor pregnancy outcomes as recommended by the WHO [15,21,22]. Although a low dose of SP has been reported to be highly effective in preventing malaria in pregnancy and reducing the consequences of malaria infection such as reduce placental malaria infection, reduction of low birth weight and severe maternal anemia [15,16,17,18,19,20], it contains sulphonamide, one of the groups of oxidant drugs capable of inducing hemolysis [2,3,23,24].

To address this, the anti-malaria drug policy for Ghana recommends that pregnant women be screened for G6PD deficiency and those with G6PD deficiency be excluded from IPTp with SP administration [22,25]. G6PD deficiency prevalence in Ghana has been estimated to range from 6.5 to 19% [26,27,28]. In view of this, it a policy of the Ghanaian Ministry of Health that the G6PD deficiency status of every pregnant woman should be checked during the first antenatal visit. However, G6PD deficiency testing in Ghana is still a laboratory-based test (microscopy) and may not be accessible to all pregnant women particularly, those accessing ANC in rural primary healthcare (PHC) clinics. Although previous studies assessed the availability and supply chain management of point-of-care (POC) test including G6PD deficiency tests [29,30,31], geographical accessibility to comprehensive ANC POC testing [32], and evaluated a POC testing device for G6PD deficiency [5], to date, no study in Ghana has assessed the geographical accessibility to POC testing for G6PD deficiency. To inform national policy on G6PD deficiency screening test, support treatment decisions with anti-malarial medicines and SP administration to pregnant women in rural PHC clinics, the national malaria control or elimination program, and to improve equity to healthcare, we mapped the geographical access (distance and travel time) to POC testing for G6PD deficiency using geographical information systems.

## 2. Methods

### 2.1. Study Design and Setting

This is a follow-up study on a prior cross-sectional study conducted to assess the accessibility of pregnancy-related POC diagnostic tests in the upper east region (UER) of Ghana [29]. Of the 100 participated PHC clinics in the earlier study, none was providing G6PD deficiency testing services in the region [29]. This current study, therefore, mapped the geographical access to POC testing for G6PD deficiency as part of ANC services. The study setting has been adequately described in the earlier published cross-sectional survey and audit [29,30]. The 2019 project population of the region is approximately 1,273,677 (51.6% females) and is considered largely (79%) rural and scattered in dispersed settlements [33].

### 2.2. Data Collection

We previously collected data on 100 randomly selected PHC clinics from all the districts in the UER as explained in the published cross-sectional survey [29]. We also collected data on all health facilities (district hospitals) providing G6PD deficiency testing in the region. To map the geographic access to G6PD deficiency testing services in UER, the geo-located data of the health facilities providing G6PD deficiency testing and that of PHC clinics were obtained from the Regional Health Directorate, and the use of global positioning system. Topographical data such as roads, rivers, and digital elevation models were obtained and juxtaposed with data obtained from the University of Ghana Remote Sensing and Geographic Information Systems laboratory to validate data accuracy. To allow for the results of spatial processes in a chosen unit of ‘meters’, the world geodetic system zone 30° N coordinate system was applied to all spatial data. PHC clinic information such as clinic type, ownership, availability of G6PD deficiency testing services, and name of nearest district hospital providing G6PD POC testing and location were obtained from the clinic managers using a questionnaire. The health facilities mentioned as accessed points were cross-checked to be sure of the availability of the G6PD deficiency testing service.

### 2.3. Variables and Operational Definitions

#### 2.3.1. Availability

The availability of health facilities providing G6PD deficiency testing services.

#### 2.3.2. Distance

The proximity from a PHC clinic/population to the nearest district hospital providing G6PD deficiency testing at point-of-care.

#### 2.3.3. Travel Time

Estimated time likely to be spent by a patient or pregnant woman traveling from a PHC clinic or any location in UER to the entrance of a district hospital for a G6PD deficiency screening test.

### 2.4. Data Analysis and Mapping

We linked the data on PHC clinics, health facilities providing G6PD deficiency screening test, area, and the geographic coordinates of the health facilities to ArcGIS 10.4 software and a base map. All 100 PHC clinics from the earlier cross-sectional survey were used as inputs to quantify the distance in kilometers (km) to their nearest health facilities providing a G6PD deficiency screening test in the study area. The euclidean distance from the PHC clinic as well as from all areas of UER to the nearest health facility was calculated using the near function analysis tools in ArcGIS 10.5. We used an assumed speed of 20 km per hour of the most available and generally utilized public transport in the region known as “motor king” (a motorized tricycle) to estimate the travel time from all areas. The model and technique used to guesstimate the travel time from all locations in UER to the closest health facilities providing G6PD deficiency screening test in this current study are published in our previous studies [32,34]. The estimated distances and travel times for each of the Districts to the nearest health facility providing G6PD deficiency testing services from PHC clinics and all locations in the UER were exported to Stata V 14.0 software for analysis and the mean and standard deviation calculated and reported. A study showed that access to healthcare beyond 10 km has an association with higher risks of poor health outcomes [35]. Therefore, this current study considered location less ≤10 km to the nearest district hospital providing G6PD deficiency screening test as zones with good geographical access.

### 2.5. Ethics Approval

This study was approved by the Navrongo Health Research Centre Institutional Review Board/Ghana Health Service (approval number: NHRCIRB291) on 8th January 2018 and the University of KwaZulu-Natal Biomedical Research Ethics Committee (approval number: BE565/17) on 12 February 2018.

## 3. Results

### 3.1. Characteristics of the Participated PHC Clinics in the Study

One hundred PHC clinics were included in this study comprising of 58% Community-based Health Planning and Services (CHPS) facilities, and 42 sub-district health centers. Of the 100 participated PHC clinics, the majority (92%) were Ghana Health Service (GHS) facilities, and the remaining 8% were Christian Health Association of Ghana (CHAG) facilities.

### 3.2. Geographical Distribution of Public Health Facilities Providing G6PD Deficiency Testing in UER

**Figure 1:**
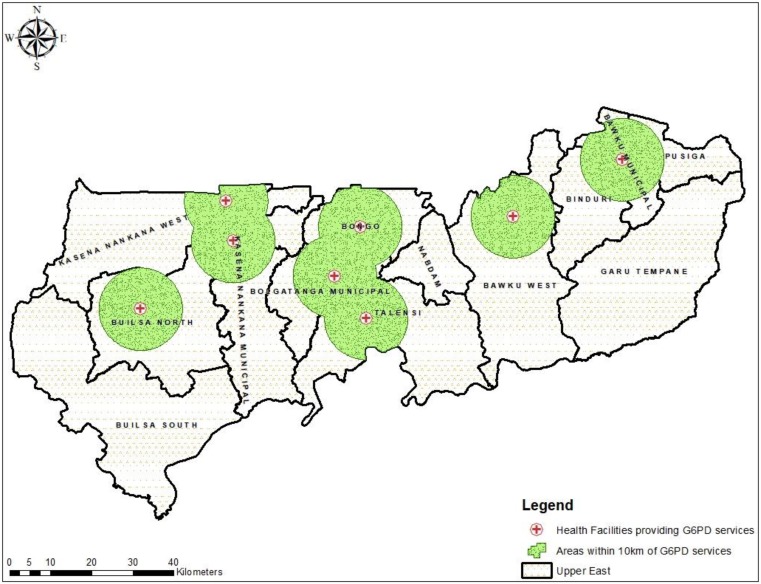
Map showing the geographic distribution of health facilities providing Glucose-6-Phosphate Dehydrogenase (G6PD) deficiency screening test and sites within 10 km distance from the health facility.

POC testing for G6PD deficiency was available in all eight hospitals. This implies that the health facility-to-population ratio for G6PD deficiency POC testing in UER was approximately 1:159,210 (8/1,273,677) population, whilst the health facility-to-women of reproductive age (WORA) was about 1:38,210 (8/305683). Of these eight hospitals, six (6) were District Hospitals owned and managed by the GHS, one (1) owned by a Church (a member of the CHAG), and one (1) Regional Hospital. Of the thirteen administrative districts in the region, five districts (Binduri, Nabdam, Garu-Tempane, Pusiga, and Builsa South) had no health facility providing G6PD deficiency POC testing, as shown in Figure 1.

### 3.3. Geographical Access to Health Facilities for G6PD Deficiency POC Testing in the UER

**Figure 2:**
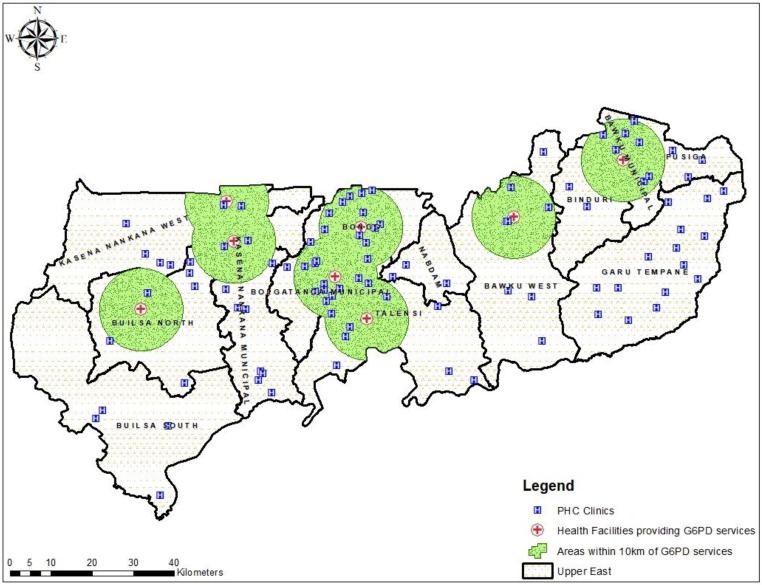
Map showing the distribution of the primary healthcare (PHC) clinics and distance within 10 km from the nearest health facility providing G6PD screening test.

We mapped the 100 randomly selected PHC clinics to the nearest hospital in the region providing G6PD deficiency testing and estimated the distance and travel time. Of the 100 PHC clinics, the majority (52%) were located in areas greater than 10 km to the nearest health facility where G6PD deficiency screening test. None of the participated clinics in three districts (Garu-Tempane, Nabdam, and Builsa South) was within 10 km proximity to the nearest health facility providing a G6PD deficiency screening test (Figure 2). The estimated mean distance from a PHC clinic to a hospital for G6PD deficiency screening test in the region was 15 ± 11 km, and the mean travel time was 46 ± 33 min based on a motorized tricycle speed of 20 km per hour.

Based on the 100 PHC clinics involved in this study, 3 districts (Bawku Municipal, Bolgatanga Municipal, and Bongo District) out of the 13 in the region recorded good geographical access (mean distance less than 10 km) to POC testing for G6PD deficiency in the region. Of the remaining ten districts that recorded poor geographical access to POC testing for G6PD deficiency, PHC clinics in the Builsa South and Garu-Tempane districts with mean distances of 30 ± 9 km (travel time; 89.6 ± 27.5 min) and 28 ± 6 km (travel time; 85 ± 18 min) respectively were shown to have poorer geographical access compared to the other districts.

**Figure 3:**
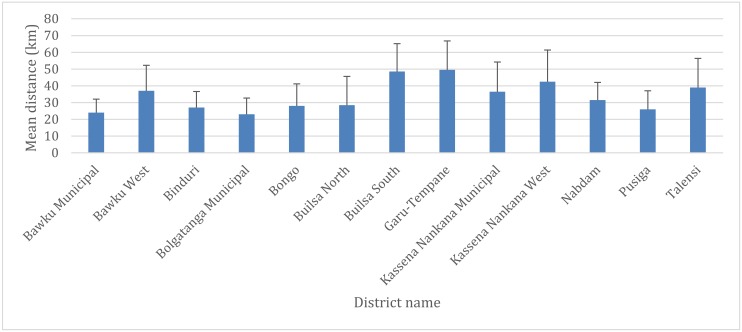
Mean distance and standard deviation from all locations to the nearest hospital providing G6PD deficiency point-of-care (POC) testing services in the upper east region (UER) by districts.

**Figure 4:**
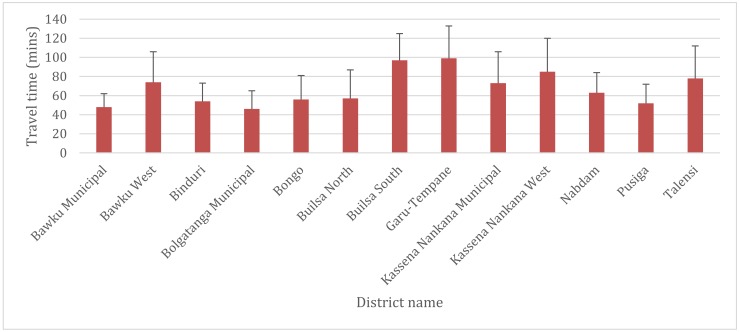
Mean travel time and standard deviation from all locations to the nearest hospital providing G6PD deficiency POC testing services in the UER by districts.

We also quantified the distance and travel time from all locations in the region to the closest health facility for G6PD deficiency screening test. The results show varied geographical access to POC testing for G6PD deficiency in all 13 districts. The results show populations in the Bolgatanga Municipality has better access to health facilities for G6PD deficiency testing [mean distance = 23 km; SD = 9, mean travel time = 46 min; SD = 19] whilst Garu-Tempane District recorded the poorest [mean distance = 50 km; SD = 17, mean travel time = 99 min; SD = 34]. The mean distance and traveling time using all locations were 34 ± 14 km and 68 ± 26 min respectively. Figure 3 and Figure 4 respectively present the mean distance and travel time to POC testing for G6PD deficiency per district in the region.

## 4. Discussion

This study revealed limited availability of health facilities offering G6PD deficiency screening test in the region. All PHC clinics lacked a POC device for G6PD deficiency screening. G6PD deficiency screening test was available in districts where a hospital was present and providing laboratory services. Moreover, the geographical access to POC testing for G6PD deficiency in the region was poor and varied across the districts.

Similarly to the present study findings, Adu-Gyasi and colleagues’ 2015 study in Ghana aimed to evaluate a G6PD deficiency POC device revealed limited availability of health facilities providing G6PD deficiency screening test [5]. Their study evidenced that no health facility except the research institution was providing G6PD deficiency screening as an essential healthcare service in their study area [5]. Elsewhere, Therrell and colleagues in 2015 reported that despite the highest prevalence of G6PD deficiency in Africa and the Middle East, these regions have the poorest access to G6PD deficiency screening tests [36]. Anderle and colleagues in their review also observed that G6PD deficiency screening services are mostly accessible to urban populations close to health facilities that provide the service [37]. Contrary to the current study findings, Ley and colleagues’ study in 2015 evidenced the availability of G6PD deficiency microscopy screening and rapid diagnostic testing (RDTs) services at all health facilities as well as community-level health facilities in Bangladesh [38]. Ley and colleagues further evinced the availability of G6PD deficiency microscopy screening and RDTs service in all hospital and clinics, and the availability of RDTs only at community-level health facilities in Cambodia [38]. Ley and colleagues study additionally showed that in the Philippines, G6PD deficiency microscopy screening was found to be available in hospitals as well as municipal health clinics [38]. Moreover, access to G6PD deficiency screening service was described to be high in the Asia Pacific region where about six countries were reported to be providing the service to the general population and sub-populations such as newborns [36].

Although we found no study reporting on the geographical access to POC testing for G6PD deficiency either in Ghana or elsewhere, previous studies that assessed the geographical access to health facilities for comprehensive ANC service, and tuberculosis POC testing in UER reported similarly poor accessibility with district variation as well as random spatial distribution of the health facilities providing the services [32,34,39]. The poor geographical accessibility to POC testing for G6PD deficiency evidenced by this current study has potential implications on Ghana’s malaria control program, screening of pregnant women prior to SP administration, and screening of newborns for G6PD deficiency as recommended by the WHO. Healthcare providers in rural PHC clinics mostly likely may be prescribing anti-malarial drugs to patients without knowledge of their G6PD deficiency status. It is also possible pregnant women particularly primigravidas (first timers) receiving ANC services in rural PHC clinics whose G6PD deficiency statuses were previously unknown may be denied SP administration unjustifiably. Traveling long distances to access G6PD deficiency screening test have financial implications, long waiting time for test results, and may result in patient dissatisfaction. Hence, patients or pregnant women may fail to go for a G6PD screening test at the referral hospital and this consequently may affect both maternal and newborn health outcomes.

**Figure 5:**
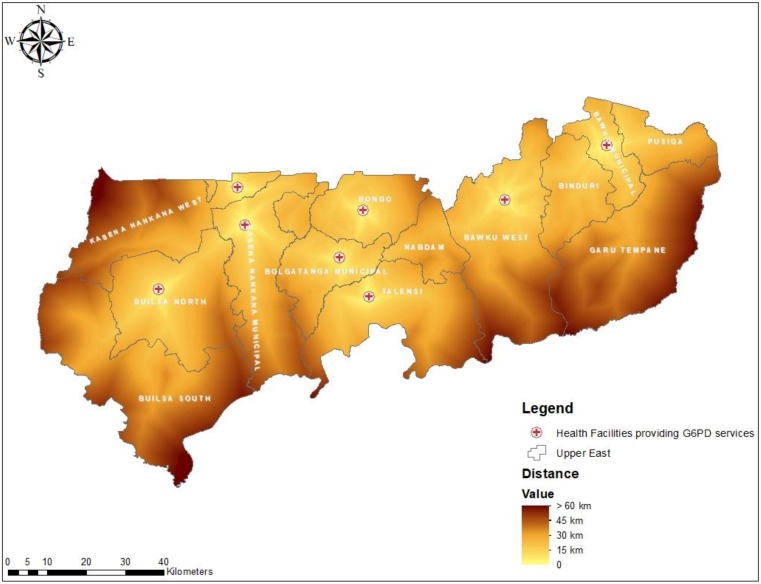
Map showing the distribution of distance (km) from all residential areas to health facilities providing G6PD screening test in the UER.

To address disparities with regards to access to G6PD deficiency testing services, inform rural healthcare providers decisions on SP or anti-malarial therapy, and improve malaria, maternal, and newborn health outcomes, we recommend the implementation of G6PD POC testing at rural PHC clinics in Ghana. For instance, locations in UER with poor geographical access to G6PD deficiency testing services as visualized in Figure 5 should be prioritized for improvement. Although the WHO has not included a G6PD deficiency screening test as one of the tests to be performed in PHC clinics in the essential list of in vitro diagnostics, several G6PD deficiency POC testing devices and RDTs are available in recent time and are have been used in countries such as Bangladesh and Cambodia [38] to improve access to G6PD deficiency testing service. Implementation of appropriate POC diagnostic tests for G6PD deficiency testing in rural and resource-limited health facilities in Ghana and other malaria-endemic countries with poor access to G6PD deficiency testing can help resolve the dilemma with the administration of SP and other antimalarial medicines health providers in those areas experience. It will also reduce the potential catastrophic indirect cost associated with accessing the service such as transportation cost, loss of man-hours at hospitals, and other unforeseen risks resulting from traveling long distances with poor transportation systems.

**Figure 6:**
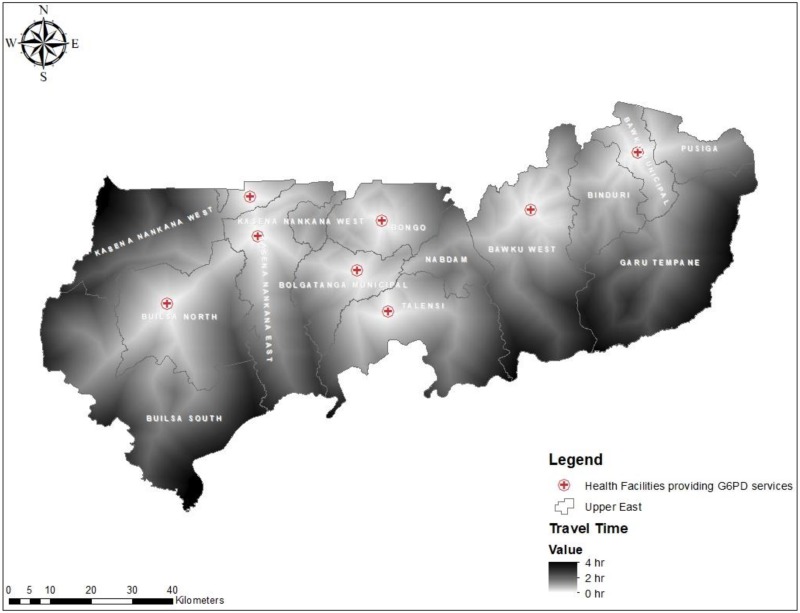
Map showing the distribution of travel time (mins) from all residential areas to health facilities providing G6PD screening test in the UER.

An additional benefit for implementing POC testing for G6PD deficiency at rural PHC clinics in UER will be a reduction of traveling time from all areas (Figure 6) and improve accessibility to the service. This may lead to increase utilization of the service and improve health outcomes. Furthermore, implementing G6PD deficiency screening tests at PHC clinics will help improve Ghana’s malaria control program as well as contribute to Ghana’s quest to attain universal health coverage. Moreover, the ability of health providers in resource-limited settings to test the G6PD deficiency status of pregnant women potentially can help them report promptly hemolytic anemia cases caused by G6PD deficiency may during pregnancy management to prevent hematological and several possible serious obstetrical complications such as fetus malformations, infertility, bilirubin-induced neurological damage in newborns, and death [6,40]. In view of these, we recommend a wider implementation of appropriate diagnostic tests for G6PD deficiency testing in rural and resource-limited settings health facilities to improve G6PD deficiency status testing of pregnant women and patients prior to the administration of SP and other antimalaria medicines as well as, facilitate the malaria control/elimination programs with primaquine in malaria-endemic countries.

Moreover, chloroquine has recently been shown to be a useful drug for the treatment of Coronavirus Disease 2019 (COVID-19) [10]. which has so far infected over 1,200,000 million people with more than 62,000 deaths [41]. Based on this, some countries are considering the use of chloroquine to treat COVID-19 cases, despite the safety concerns raised regarding its use due to the possibility of chloroquine causing hemolysis in patients with G6PD deficiency [9]. Considering this, checking the G6PD deficiency status of a COVID-19 patient prior to treatment with chloroquine may be ideal, particularly in malaria-endemic countries such as Ghana. Hence, it would be prudent to improve the availability of and accessibility to POC testing for G6PD deficiency prior to the recommendation of the use of chloroquine for treatment of COVID-19 patients in malaria-endemic countries such as Ghana.

Using GIS to inform the implementation of health services has been shown to be useful [42,43,44]. GIS effectively enables the implementation of POC testing in health networks to streamline decision making at the POC [45]. Geospatial science has also been proven to be useful in creating solutions for population access to POC testing during emergencies, outbreaks, and disasters such as the present COVID-19 pandemic [46]. Hence, GIS helps improve patient outcomes, time and money, ensure that the health networks are sufficiently resourced to deliver needed health services to the population [45]. Despite these strengths, there are several limitations worth noting such as our inability to include non-spatial factors in the analysis. Non-spatial factors such as the income of a patient [47], age, cultural practices, education, and others can also influence the utilization of a health service even if the service is very close to the individual [48]. Although this study is recommending implementation of POC testing for G6PD deficiency at rural PHC clinics, it did not assess the cost-benefit analysis of its implementation. However, knowing the G6PD status of a patient is useful clinical information and the cost of implementing POC for G6PD deficiency testing service at rural PHC clinics by the Government of Ghana should not be a reason to deny a special sub-population such as pregnant women and newborns access to G6PD deficiency testing because Ghana is a malaria-endemic country. It is also possible some private hospitals and medical laboratories within the region may be providing G6PD deficiency screening test and where not included in this study’s analysis. The travel time estimation provided by this study was dependent on only one mode of public transport using an assumed speed which might be inaccurate. Moreover, this study was only limited to only one region in Ghana and therefore the findings may not necessarily apply to the remaining fifteen regions in the country. We, therefore, recommend future researches to focus on areas such as the non-spatial factors, cost-benefit analysis of implementing POC testing for G6PD deficiency at PHC clinics not captured by this study. We also recommend a similar study in the other fifteen regions of Ghana. We further recommend future studies to included other modes of transportation such as walking, car, motorbike, bicycle, and others where applicable in the analysis. Nonetheless, this study has provided evidence-based information useful for policy decision making for targeted improvement of POC testing for G6PD deficiency in the UER. This study also provides baseline information for future studies since it is the first study to evaluate the geographical accessibility to POC testing for G6PD deficiency in Ghana and perhaps, worldwide.

## 5. Conclusions

G6PD deficiency POC test permits clinical diagnostic decisions either to administer anti-malarial drugs/SP or not in resource-limited settings and useful for improving health outcomes and the cost of accessing G6PD testing services. Spatial analysis estimated current mean distance and travel to a G6PD deficiency testing center from all locations in UER to be over 33 km and 60 min respectively using a motorized tricycle. This could be reduced significantly with the implementation of POC testing for G6PD deficiency at PHC clinics located more than 10 km away from a health facility. Given the challenges associated with G6PD deficiency, it would be essential to improve access to G6PD deficiency POC testing to facilitate administration of sulphadoxine-pyrimethamine to pregnant women, full implementation of the malaria control program in Ghana, and treatment of COVID-19 patients with chloroquine in malaria-endemic countries. To enable the World Health Organization include appropriate G6PD POC diagnostic tests in its list of essential in-vitro diagnostics for use in resource-limited settings, we recommend a wider evaluation of available POC diagnostic tests for G6PD deficiency, particularly in malaria endemic countries.

## Abbreviations

ANC	Antenatal care
COVID-19	Coronavirus Disease 2019
G6PD	Glucose-6-Phosphate Dehydrogenase
GIS	Geographic Information Systems
PHC	Primary Healthcare
POC	Point-of-Care
RDT	Rapid Diagnostic Test
SDGs	Sustainable Development Goals
UER	Upper East Region
WHO	World Health Organization
